# Inhibition of Protein Glycation by Tiger Milk Mushroom [*Lignosus rhinocerus* (Cooke) Ryvarden] and Search for Potential Anti-diabetic Activity-Related Metabolic Pathways by Genomic and Transcriptomic Data Mining

**DOI:** 10.3389/fphar.2018.00103

**Published:** 2018-02-14

**Authors:** Hui-Yeng Y. Yap, Nget-Hong Tan, Szu-Ting Ng, Chon-Seng Tan, Shin-Yee Fung

**Affiliations:** ^1^Department of Oral Biology, Faculty of Dentistry, Mahsa University, Kuala Lumpur, Malaysia; ^2^Medicinal Mushroom Research Group, Department of Molecular Medicine, Faculty of Medicine, University of Malaya, Kuala Lumpur, Malaysia; ^3^LiGNO Biotech Sdn Bhd, Balakong Jaya, Malaysia; ^4^Center for Natural Products Research and Drug Discovery, University of Malaya, Kuala Lumpur, Malaysia

**Keywords:** *Lignosus rhinocerus*, tiger milk mushroom, terpenoid biosynthesis, anti-glycation, superoxide anion radical scavenging

## Abstract

Naturally occurring anti-glycation compounds have drawn much interest in recent years as they show potential in reducing or preventing the risk of chronic complications for diabetic patients. In this study, annotation of the genome–transcriptome data from tiger milk mushroom (*Lignosus rhinocerus*, syn. *Lignosus rhinocerotis*) to PlantCyc enzymes database identified transcripts that are related to anti-diabetic properties, and these include genes that are involved in carotenoid and abscisic acid biosynthesis as well as genes that code for glyoxalase I, catalase-peroxidases, and superoxide dismutases. The existence of these genes suggests that *L. rhinocerus* may contain bioactive compound(s) with anti-glycation properties that can be exploited for management of diabetic complications. A medium-molecular-weight (MMW) fraction which was obtained from a combination of cold water extraction and Sephadex^®^ G-50 (fine) gel filtration chromatography of *L. rhinocerus* sclerotia powder was demonstrated to exhibit potent anti-glycation activity. The fraction specifically inhibited the formation of N𝜀-(carboxymethyl)lysine, pentosidine, and other advanced glycation end-product (AGE) structures in a human serum albumin-glucose system, with an IC_50_ value of 0.001 mg/ml, almost 520 times lower than that of the positive control, aminoguanidine hydrochloride (IC_50_ = 0.52 mg/ml). Its ability to suppress protein glycation may be partly associated with its strong superoxide anion radical scavenging activity (10.16 ± 0.12 mmol TE/g). Our results suggest that the MMW fraction of *L. rhinocerus* shows potential to be developed into a potent glycation inhibitor for preventing AGE-mediated diabetic complications.

## Introduction

The Polyporale tiger milk mushroom [*Lignosus rhinocerus* (Cooke) Ryvarden; syn. *Lignosus rhinocerotis*] is an important medicinal mushroom that has been used as a remedy to treat various health problems including wounds, fever, whooping cough, asthma, food poisoning, chronic hepatitis, gastric ulcers, and cancer (Jones et al., 2007; Wong and Cheung, 2009). Traditional consumption of its sclerotium (the part with medicinal value) is usually in the form of cold water extract. The sclerotium is first grated on a hard surface (e.g., granite plate) in the presence of water and the moist grated product is added to more water (Chan, 1953). In other instances, the sclerotium has been reported to be sliced, boiled, and consumed in the form of a decoction (Chang and Lee, 2004; Azliza et al., 2012). The sclerotial extracts of *L. rhinocerus* have been reported to exhibit anti-hypertensive, anti-inflammatory, anti-proliferative, neurite outgrowth, and antimicrobial activities along with the enhancement of immunomodulatory activity (Lai et al., 2008; Wong et al., 2011; Eik et al., 2012; Lee et al., 2012; Shopana et al., 2012). Antioxidant capacity of *L. rhinocerus* has also been investigated. Suziana Zaila et al. (2013) reported a weak antioxidant capacity of alkaloids-containing pressurized liquid extracts from wild *L. rhinocerus* sclerotium by ferric reducing antioxidant power (FRAP) assay, whilst Lau and colleagues described the free-radical scavenging activities, reducing properties, metal chelating activities, and inhibitory effects on lipid peroxidation of *L. rhinocerus* (KUM61075) aqueous methanol extracts from different morphological/developmental stages (mycelium and sclerotium) and culture conditions (shaken and static cultures) of liquid fermentation (Lau et al., 2014). We have also demonstrated that the cold water extract of *L. rhinocerus* TM02 cultivar’s sclerotia exhibited strong superoxide anion radical (${\text{O}}_{2}^{•-}$) scavenging activity with potency comparable to rutin (Yap et al., 2013). Elucidation of *L. rhinocerus* genome provided a better insight into its biology and brought new prospects for exploration (Yap et al., 2014). Yeast-based genome mining guided by transcriptomics approach which has been reported recently has enabled the discovery of several bioactive sesquiterpenoids, namely (+)-torreyol and α-cadinol from this mushroom (Yap et al., 2017).

Advanced glycation end-products (AGEs) is a chemically heterogeneous group of compounds comprising of N𝜀-(carboxymethyl)lysine (CML), carboxymethyl-hydroxylysine (CMhL), pentosidine (Pent), pyrraline, crossline, 2-(2-furoyl)-4(5)-(2-furanyl)-1H-imidazole, and others. These compounds are capable of increasing the oxidative damage to cells and altering their normal functions. The pathological effects of AGEs are associated with inflammation and oxidative stress, which can lead to cellular disorders in biological systems (Eble et al., 1983; Schmidt et al., 1999). *In vivo* accumulation of AGEs has been described as the hallmark of pathogenic chronic diabetic complications such as retinopathy, neuropathy, nephropathy, and other health disorders such as atherosclerosis and aging (Gkogkolou and Bohm, 2012; Perez Gutierrez, 2012). Maillard reaction drives the formation of stable Amadori compounds through a series of *in vivo* rearrangements, and are subsequently converted to a number of reactive intermediates capable of forming AGEs (Bucala and Cerami, 1992). When glycation (or non-enzymatic glycosylation) is accompanied by oxidation, the process is known as “glycoxidation”. CML and Pent are examples of products of glycoxidation (Singh et al., 2001; Ulrich and Cerami, 2001). Reactive dicarbonyl compounds or α-oxoaldehydes such as methylglyoxal and 3-deoxyglucosone (3-DG) are important precursors of AGEs *in vivo* and intermediate products formed during Amadori rearrangement (Baynes, 1991; Wells-Knecht et al., 1996). Both products have been shown to induce oxidative stress and apoptosis in macrophage-derived cell lines (Okado et al., 1996).

Glycation which may lead to AGEs-induced toxicity is known to be associated with increased free-radical production (Kazeem et al., 2012). Therefore, searches for effective AGE inhibitors are often from compounds with good antioxidative properties (Price et al., 2001; Gkogkolou and Bohm, 2012). It was reported that superoxide radical scavengers could alleviate oxidative stress and reduce the generation of reactive carbonyl compounds (Baynes, 1991; Wu et al., 2011). Hence, we hypothesized that *L. rhinocerus* may possess anti-glycation potential that could be medically exploited in view of its strong ${\text{O}}_{2}^{•-}$ scavenging ability (Yap et al., 2013). Numerous exogenous antioxidants found in vitamins and foods are able to inhibit AGEs generation indirectly by scavenging free radicals formed during glycation and preventing the reduction of sugars and Amadori products from self-oxidation (Elosta et al., 2012). As an example, spices and herbs including ginger, cinnamon, cloves, marjoram, rosemary, and tarragon has been shown to inhibit glycation of albumin *in vitro* due to high phenolic composition (Dearlove et al., 2008). Yan et al. (2013) reported the *in vitro* anti-glycation and antiradical activities of polysaccharides isolated from the mycelium of a mushroom (*Ganoderma capense*). The investigation of mushrooms with good AGEs inhibitory activities could lead to the discovery of new therapeutic agent(s) for the prevention of diabetic complications.

In this study, we mined several molecular signatures (transcripts) of interest in this study via pathway annotation from the *L. rhinocerus* genome and transcriptome datasets (Yap et al., 2014, 2015a). We also report the glycation inhibitory activity of the sclerotial cold water extract of *L. rhinocerus* TM02 cultivar and pooled fractions of the extract obtained from Sephadex^®^ G-50 (fine) gel-filtration chromatography. The phytochemical content, Trolox-equivalent antioxidant-capacity assay (TEAC), and FRAP of the fractions were also evaluated to compare their anti-glycation capacities to the phytochemical content and antioxidant activities, in particular the ${\text{O}}_{2}^{•-}$ scavenging activity.

## Materials and Methods

### Gene Functional Annotation and Expression Study

Predicted genes from the genome of *L. rhinocerus* (Yap et al., 2014) were functionally annotated based on homology to Plant Pathway information [PlantCyc enzymes database v2.0^1^, downloaded as on 15th June 2016]. Sequence similarity (“homology”) search was performed using locally installed blast-2.4.0+ software (BLASTP cut-off e-value ≤1e-5). The *L. rhinocerus* genome (AXZM01000000) from DDBJ/EMBL/GenBank was used. Gene expression level was determined by the reads per kilobase per million reads (RPKM) method developed by Mortazavi et al. (2008) to eliminate the influence of different gene lengths and sequencing discrepancies, as reported in previous study with the accession number SRR1509475 under experiment SRX648275 (Yap et al., 2015a).

### Fractionation of *L. rhinocerus* Sclerotial Extract

Freeze-dried 0.2 mm sieved sclerotial powder of *L. rhinocerus* TM02 cultivar was obtained from LiGNO Biotech Sdn Bhd (Balakong Jaya, Selangor, Malaysia). The product was authenticated by DNA fingerprinting as previously described where the internal transcribed spacer regions of ribosomal RNA of the mushroom was sequenced (Tan et al., 2010). Sclerotial powder was extracted with water at 4°C by 24-h stirring in a mass-to-volume ratio of 1:20 (g/ml) followed by centrifugation (8,000 ×*g*, 30 min) and filtration using Whatman^®^ qualitative filter paper, Grade 1. The extract was freeze-dried and stored at -20°C until further use. Fractionation of the cold water extract was performed via Sephadex^®^ G-50 (fine) gel-filtration chromatography. Elution was carried out by gravity and a total of 100 tubes (2.5 ml/tube) were collected. Molecular mass of the pooled fractions was determined by comparing their ratio of elution volume (V_e_) and void volume (V_o_) to the V_e_/V_o_ of selected protein standards including carbonic anhydrase (29 kDa), soybean trypsin inhibitor (20 kDa), ribonuclease A (14 kDa), cardiotoxin (7 kDa), cobalamin (1.35 kDa), and riboflavin (376 Da).

### Total Carbohydrate and Protein Content Determinations

Carbohydrate and protein contents were determined with phenol–sulfuric acid method as described by Dubois et al. (1951) and Bradford method (Bradford, 1976), respectively. D-Glucose and bovine serum albumin (BSA) were used as standards, respectively, for carbohydrate and protein determinations.

### Total Phenolic and Terpenoid Content Determinations

Total phenolic content was determined by Folin-Ciocalteu method as described previously, using gallic acid as standard (Yap et al., 2013). Phenolic content of the fractions was expressed as mg gallic acid equivalents (GAE). Total terpenoid content was estimated according to Ghorai et al. (2012) using linalool as standard. Terpenoid content of the fractions was expressed as mg linalool equivalents (LE).

### Antioxidant Assays

Antioxidant assays were adapted from our previous study (Yap et al., 2013). FRAP assay was carried out according to Benzie and Strain (1996) using ferrous sulfate (FeSO_4_) as standard. The 1,1-diphenyl-2-picrylhydrazyl (DPPH^•^), 2,2-azinobis-3-ethylbenzothiazoline-6-sulfonic acid (ABTS^•+^), and ${\text{O}}_{2}^{•-}$ scavenging activities of the fractions were determined according to Re et al. (1999), Cos et al. (2002), and Siddhuraju and Becker (2007), respectively, using Trolox as standard. Antioxidant activity measurement was expressed as TEAC in mmol Trolox equivalents (TE). Quercetin and rutin served as positive controls.

### Human Serum Albumin (HSA)-Glucose Glycation Assay

The glycation (Maillard reaction) *in vitro* assay was modeled according to Yonei et al. (2008) with slight modification. HSA-glucose assay is used to evaluate the ability of the sample to inhibit the glucose-mediated protein glycation. Reaction mixture consisting of 0.05 mol/L PBS (pH 7.4), 8 mg/ml HSA, and 0.2 mol/L glucose solution was incubated in a water bath set to 60°C for 40 h to generate AGEs in a 1.5-ml tube. Sodium azide of 0.2 g/L was added to prevent contamination. Sample was added at desired concentration. Aminoguanidine hydrochloride (AG), a known AGE inhibitor, was used as positive control. Concentration of AGEs was quantified as described in Section “Quantification of Advanced Glycation End-Products (AGEs)” and percentage inhibition rate of AGEs generation was determined by using the formula (1 – (A – B)/(C – D)) × 100 where A is the amount of AGEs in the test sample containing extracts or AG with glucose solution; B is the amount of AGEs in the test sample containing extracts or AG with distilled water instead of glucose solution; C is the amount of AGEs in the test sample without extracts or AG with glucose solution added and incubated; and D is the amount of AGEs in test sample without extracts, AG, or glucose solution. A graph of the AGE-inhibition rate was plotted to determine the half maximal inhibitory concentration (IC_50_).

### BSA-Methylglyoxal Glycation Assay

Bovine serum albumin-methylglyoxal assay was carried out according to Perez Gutierrez (2012) to evaluate the middle stage of protein glycation. BSA and methylglyoxal in 100 mM PBS (pH 7.4) were dissolved to a concentration of 20 mg/ml and 60 mM, respectively. Sample (at 5 mg/ml) in the same PBS buffer was added in that sequence in a ratio of 1:1:1 in a 1.5 ml tube. Reaction mixture was incubated at 37°C for 7 days to generate AGEs. Sodium azide of 0.2 g/L was used as the aseptic agent. AG served as positive control. Concentration of fluorescent AGEs was quantified as described in Section “Quantification of Advanced Glycation End-Products (AGEs)” and percentage inhibition of AGEs generation was determined using the formula as described in Section “Human Serum Albumin (HSA)-Glucose Glycation Assay” with slight modification(s).

### Quantification of Advanced Glycation End-Products (AGEs)

Advanced glycation end-products-derived fluorescence was measured at an excitation wavelength of 370 nm and an emission wavelength of 445 nm. Non-fluorescent AGEs were quantified using OxiSelect^TM^ Advanced Glycation End Product (AGE) Enzyme-linked Immunosorbent Assay (ELISA) Kit (Cell Biolabs, Inc., San Diego, CA United States) according to manufacturer’s product manual using AGE-BSA as standard. The AGE ELISA Kit uses an anti-AGE polyclonal antibody that detects multiple AGE structures including CML and Pent, but not N𝜀-(carboxyethyl)lysine (CEL) and methylglyoxal.

### Statistical Analysis

Results were expressed as mean ± standard deviation (SD) of at least two independent experiments which were performed in triplicates, unless otherwise stated. Statistical Package for the Social Sciences (SPSS) version 17.0 (SPSS, Inc., Chicago, IL, United States) with one-way ANOVA followed by the LSD’s *post hoc* test for multiple comparisons was used to compare mean values. A *p*-value of less than 0.05 was considered as statistically significant.

## Results and Discussion

### Functional and Pathway Annotations

**FIGURE 1:**
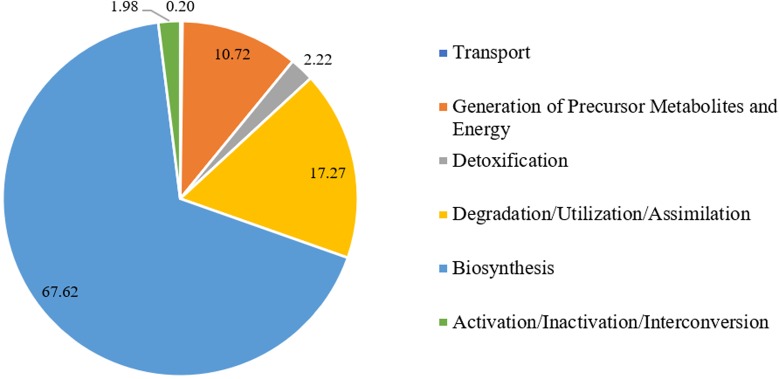
Percentage distribution of *Lignosus rhinocerus* transcripts obtained from PlantCyc enzymes annotation in the first layer.

The PlantCyc reference database was used in this study to uncover gene(s) of interest that might be relevant to the bioactivity, in particular the glycation inhibitory potential of *L. rhinocerus*. Annotation to the database merely covered 26.08% [2801 non-redundant (mRNA) transcripts] of the 10,742 putative genes from *L. rhinocerus* sequenced genome (**Supplementary Data Sheet 1**) due to genetic distance. Among them, 1291 transcripts were annotated to a variety of metabolic pathway classes, which were further divided into subclasses and groups based on: (1) biological functions and (2) classes of metabolites produced and/or consumed (**Supplementary Data Sheet 1**). The pathways from PlantCyc enzymes annotation are assigned into seven layers where the first layer consists of 10 main sections, including “Activation/Inactivation/Interconversion (31 instances)”, “Biosynthesis (915 instances)”, “CDP-diacylglycerol biosynthesis IV”, “Degradation/Utilization/Assimilation (248 instances)”, “Detoxification (21 instances)”, “Generation of Precursor Metabolites and Energy (63 instances)”, “Macromolecule Modification (2 instances)”, “Metabolic Clusters (35 instances)”, “Superpathways (119 instances)”, and “Transport (12 instances)” which are further divided into several small entries/instances (layers). The majority of transcripts are involved in “Biosynthesis” followed by “Degradation/Utilization/Assimilation” and “Generation of Precursor Metabolites and Energy” at 67.62% (2013), 17.27% (514), and 10.72% (319), respectively (**Figure 1**).

**FIGURE 2:**
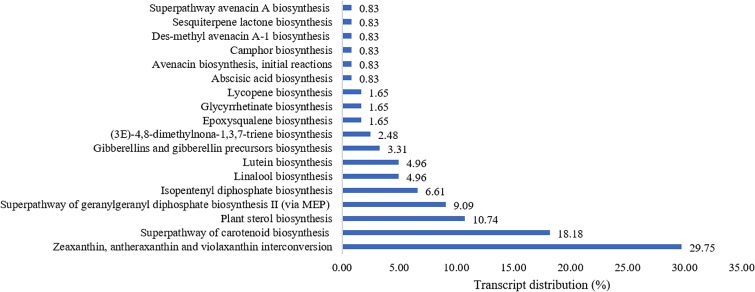
Percentage distribution of terpenoid biosynthesis pathway-related transcripts observed from PlantCyc enzymes annotation (Level 4).

The terpenoid pathways and their links to other secondary metabolite pathways (**Figure 2**) were further examined to investigate the underlying factor contributing to the medicinal properties of *L. rhinocerus*. A large number of transcripts responsible for zeaxanthin, antheraxanthin, and violaxanthin interconversion in carotenoid (also known as tetraterpenoid) biosynthesis were observed and this was followed by the biosynthesis of other types of terpenes and terpenoids including hemiterpenes, sesqui-, mono-, di-, and triterpenoids. This was not unexpected, as previous study on *L. rhinocerus* transcriptome analysis revealed a few highly expressed gene clusters that encode for sesquiterpene synthases which may be responsible for the production of several sesquiterpene alcohols (C_15_H_26_O), including cadinols and germacrene D-4-ol (Yap et al., 2015a, 2017).

Plants use mevalonic acid pathway (MVA) and the plastidic methylerythritol 4-phosphate (MEP) to produce isopentenyl diphosphate (IPP) and dimethylallyl diphosphate (DMAPP) as the basic building blocks for carotenoids. A series of condensation reactions of IPP and DMAPP will lead to the formation of geranylgeranyl pyrophosphate (GGPP) that can be converted into carotenes or xanthophylls by undergoing cyclization and/or chemical modifications within the carotenoid biosynthetic pathway (Nisar et al., 2015). Although terpenoids are predominantly produced via the MVA pathway in fungi, we found that *L. rhinocerus* also expressed genes that are involved in the biosynthesis of GGPP through the MEP pathway (**Figure 2**). Fungal carotenoids are believed to have no apparent role physiologically in the fungal cells owing to their absence in some species but instead, they appear to provide non-essential functions associated with stress tolerance and/or physiologically active by-product synthesis. Their presence may offer protection from oxidative stress and exposure to visible light or UV irradiation (Avalos and Carmen Limon, 2015). Carotenoids like lycopene and astaxanthin have been shown to positively influence the cardiovascular system with their hypolipemic properties (Kulczyński et al., 2017). Meanwhile, xanthophylls (carotenoids with molecules containing oxygen) such as lutein and zeaxanthin have been shown to be involved in the reduction of cataract and macular degeneration (Bernstein et al., 2016). Identification of transcripts (4.96%) related to pathways of lutein biosynthesis in *L. rhinocerus* (**Figure 2**) may therefore have important implication on the presence of such benefits in the mushroom itself. However, further study is warranted. Carotenoids may be cleaved to apocarotenoids which include vitamin A derivatives, the plant hormone abscisic acid, and bixin where the latter has been shown to possess anti-diabetic activity. We also found transcripts for abscisic acid biosynthesis in *L. rhinocerus* which is related to anti-diabetic, anti-obesity, and immunomodulation function(s) (Annadurai et al., 2012). Abscisic acid was reported to be an endogenous stimulator of insulin release from human pancreatic islets with cyclic ADP ribose as second messenger (Bruzzone et al., 2008).

Accumulation of harmful reactive species may contribute to many metabolic disorders and oxidative stress that has been implicated in the pathogenesis of a wide variety of diseases including diabetes mellitus, particularly the type 2 diabetes (Asmat et al., 2016). Although clinical evidence on the beneficial effect(s) of natural antioxidants ingestion and/or dietary supplementation in diabetes management is yet to be established, antioxidant therapy remains encouraging so long as the criteria for the identification of potential candidates for antioxidant be improved (Johansen et al., 2005; Bajaj and Khan, 2012). Interestingly, carotenoids are known to be efficient scavengers of singlet molecular oxygen as well as peroxyl radicals and they may act synergistically with other antioxidants present in the cell (Stahl and Sies, 2003; Skibsted, 2012). In addition to the number of transcripts involved in carotenoids synthesis, *L. rhinocerus* also has genes that are able to reduce the oxidative damage in cells through the superoxide radical degradation pathway by acting as catalase (CAT)-peroxidases and/or superoxide dismutases (SODs). In fact, both enzymes are good biomarkers of oxidative stress in diabetes mellitus and they play crucial roles in the conversion of superoxide radicals (or hydrogen peroxide) to oxygen and water. SODs are encoded by GME2619_g, GME6099_g, and GME7293_g while GME1349_g, GME5487_g, and GME6571_g code for CATs. These genes in addition to those involved in abscisic acid and lutein biosynthesis are shown in **Table 1** and the genes’ CDS sequences are provided in **Supplementary Data Sheet 2** in FASTA format.

**Table 1:** List of notable genes in *L. rhinoceros.*

Gene ID	Gene description (PlantCyC)	RPKM	e-Value	Pathway	Conserved domain(s)
GME1349_g	Catalase-peroxidase	0.670	2.89E-144	Superoxide radical degradation	
GME2619_g	Superoxide dismutase	1.930	3.53E-75		
GME5487_g	Catalase-peroxidase	1.168	3.2E-120		
GME6099_g	Superoxide dismutase	1.407	6.64E-18		
GME6571_g	Catalase-peroxidase	3.397	1.02E-174		
GME7293_g	Superoxide dismutase	2.227	2.87E-46		
GME6464_g	MLOC_3876.1	1.021	2.31E-18	Lutein biosynthesis	CypX, p450, PLN02426
GME1778_g	LOC_Os02g07680.1	1.740	1.36E-22		CypX, p450, PLN02738
GME977_g	Beta-ring hydroxylase	-0.010	0.0000019		CypX, p450, PLN02738
GME4013_g	Xanthoxin dehydrogenase	1.238	1.3E-29	Abscisic acid biosynthesis	
GME3047_g	Xanthoxin dehydrogenase	1.441	2.79E-22		
GME3225_g	Lactoylglutathione lyase	2.551	2.45E-50	—	GlxI_Zn, PLN03042, glyox_I, Glyoxalase, GloA

*RPKM, reads per kilobase per million reads.*

From **Table 1**, GME6464_g, GME1778_g, and GME977_g that code for respective MLOC_3876.1, LOC_Os02g07680.1, and beta-ring hydroxylase are cytochromes P450 (CYPs) that belong to the p450 superfamily. Each gene carries a CypX domain that plays a role in defense mechanism, transport and catabolism, and secondary metabolites biosynthesis (specifically the lutein biosynthesis). GME6464_g further carries a PLN02426 domain that is exclusive to CYP family 94 subfamily C proteins, indicating its similarity to the latter. Meanwhile, GME1778_g and GME977_g are carotene beta-ring hydroxylases that carry the PLN02738 domain. This enzyme catalyzes the C3 hydroxylation of the beta ring of a carotene as precursors to produce dihydroxy xanthophylls like lutein and zeaxanthin (Tian et al., 2003). It is to be noted that GME977_g has a rather large e-value with negative expression. Therefore, its identity remains questionable. On the other hand, GME4013_g and GME3047_g encode for xanthoxin dehydrogenase, which catalyzes the conversion of xanthoxin to abscisic aldehyde, which is an intermediate in the biosynthesis of abscisic acid.

We also found a transcript, namely GME3225_g, that encodes for lactoylglutathione lyase (or more commonly known as glyoxalase I; EC 4.4.1.5) in the genome of *L. rhinocerus* at considerable expression level. GME3225_g contains GlxI_Zn, PLN03042, glyox_I, Glyoxalase, and GloA domains that belong to the vicinal oxygen chelate (VOC) superfamily. Glyoxalase I is a critical enzyme in methylglyoxal detoxification that catalyzes the conversion of hemimercaptal which is formed from methylglyoxal and glutathione to *S*-lactoylglutathione. Therefore, this enzyme has been shown to play a critical role in the enzymatic defense against α-oxoaldehyde-mediated glycation that appears to be a factor associated with risk of developing vascular complications of uremia as well as diabetes (Thornalley, 2003). Hitherto, transcript identification by annotating the genome–transcriptome data of *L. rhinocerus* to PlantCyc enzymes database provided us insights into their possible applications and the potential beneficial properties of the mushroom by referring to available literature. These data will be valuable to establish the connection between genes and molecules for compound isolation work in pharmacological and industrial applications. Future work could also include recombinant expression and characterization. In view of the presence of several annotated genes in *L. rhinocerus* that may have important implications for antioxidant activity and anti-diabetic potential, we next determined the total phenolic and terpenoid contents, anti-glycation, and antioxidant properties of fractionated *L. rhinocerus* sclerotial cold-water extract *in vitro*.

### Fractionation of *L. rhinocerus* Sclerotial Cold-Water Extract

**FIGURE 3:**
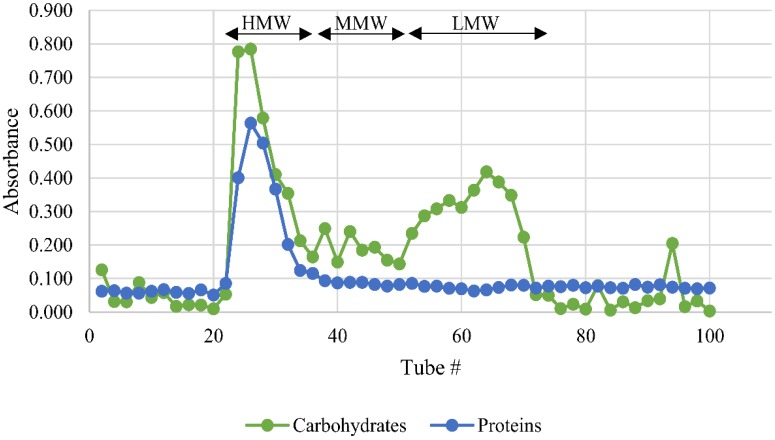
Sephadex^®^ G-50 fractionation of *L. rhinocerus* TM02 sclerotial cold-water extract. A total of 100 tubes (2.5 ml/tube) were collected. The carbohydrate and protein contents of each tube were determined and expressed as absorbance at 490 and 595 nm, respectively. The tubes were pooled into three fractions based on their molecular weight-range namely HMW, MMW, and LMW for high-molecular-weight, medium-molecular-weight, and low-molecular-weight fractions, respectively. HMW contained the bulk of proteins and carbohydrates. LMW contained low-molecular-weight carbohydrates and a small amount of proteins. MMW were constituted of the tubes between the HMW and LMW fractions.

Cold-water extraction of *L. rhinocerus* sclerotial powder yielded 20.77 ± 1.30% of freeze-dried solid. Further fractionation of the extract by Sephadex^®^ G-50 gel-filtration chromatography yielded a broad protein peak and several carbohydrate peaks (**Figure 3**). The collected tubes were pooled into three fractions based on their molecular weight range. They are the high-, medium-, and low-molecular-weight fractions for HMW, MMW, and LMW, respectively.

**Table 2** shows the yield, chemical composition, and the estimated molecular weight range of the three pooled fractions. HMW, with molecular weight range of more than 30 kDa, contains the highest amounts of carbohydrates and proteins, thus suggesting the existence of polysaccharide-protein complexes in the fraction. LMW has considerably lower carbohydrate content with very little proteins, but it has a higher content of secondary metabolites such as phenolics and terpenoids. The quantitative colorimetric-based assay adapted from Ghorai et al. (2012) is however just an estimation as the absorbance could as well be influenced by the presence of other low-molecular-weight secondary metabolite(s) such as phenolics and/or alkaloids that reacted with sulfuric acid and exhibited a similar spectrum as terpenoids. A more accurate quantitation can be carried out by gas chromatography-mass spectrometry (MS) analysis. Meanwhile, the carbohydrate and protein contents of HMW and LMW were found to be comparable to those reported in the earlier study by Lee et al. (2012). MMW has moderate amounts of the proteins, carbohydrates, and secondary metabolites with molecular weight ranging from 7.1 to 27.4 kDa. Previous study has elucidated the protein composition of selected anion exchange chromatographic fractions from MMW by liquid chromatography (LC)–MS analysis and it was found that this fraction partially contains cytotoxic serine protease(s), immunomodulatory proteins, metalloproteases, aegerolysin-domain-containing proteins, several variants of ubiquitin family, and hypothetical proteins (Yap et al., 2015c).

**Table 2:** Yield, chemical composition, and molecular weight range of Sephadex^®^ G-50 fractions from *L. rhinocerus* TM02 sclerotial cold-water extract.

	HMW	MMW	LMW
Yield (mg/g DW)	300	200	500
MW range (kDa)	>29.9	7.1–27.4	0.8–6.5
Protein (%)	3.63 ± 0.26^a^	0.76 ± 0.10^b^	0.28 ± 0.03^c^
Carbohydrate (%)	71.70 ± 5.48^a^	32.51 ± 5.00^b^	21.62 ± 8.13^b^
Phenolics (mg GAE/g)	4.05 ± 0.50^a^	4.87 ± 0.43^b^	9.35 ± 0.84^c^
Terpenoids (mg LE/g)	87.70 ± 12.85^a^	339.51 ± 13.97^b^	1,179.65 ± 65.55^c^

*Yield is expressed on a dry weight (DW) basis. Total phenolic and terpenoid contents are expressed as mg gallic acid equivalents (GAE) and mg linalool equivalents (LE), respectively. Data expressed as mean ± SD (n = 3). Means in the same row with different letters (a–c) are significantly different (p < 0.05). MW, molecular weight; HMW, high-molecular-weight fraction; MMW, medium-molecular-weight fraction; LMW, low-molecular-weight fraction.*

### Antioxidant Activities of the Sephadex^®^ Fractions

The antioxidant potential of the Sephadex^®^ G-50 fractions was evaluated by their reducing power and free radical-scavenging abilities (**Table 3**). The ferric reducing power is expressed in term of initial rate (μmol/min/g) as FRAP value. A 0–4 min reaction-time window is often appropriate for measurement (Benzie and Strain, 1996). In comparison to the positive controls quercetin and rutin, the fractions demonstrated a continued but much slower increase in reaction to reduced ferric-tripyridyltriazine (Fe^3+^-TPTZ) to ferrous complex (Fe^2+^-TPTZ) within the specified time frame, indicating some rather weak reducing capabilities. It has been suggested that dietary polyphenols react more slowly and thus require longer reaction times (≥30 min) for total quantification (Pulido et al., 2000).

**Table 3:** Antioxidant activities of Sephadex^®^ G-50 fractions from *L. rhinocerus* TM02 sclerotial cold-water extract.

	TEAC (mmol TE/g)			FRAP value (μmol/min/g)
	DPPH^•^	ABTS^•+^	${\text{O}}_{2}^{•-}$	
HMW	nil	0.31 ± 0.02^a^	7.36 ± 0.12^a^	0.25 ± 0.00^a^
MMW	nil	0.49 ± 0.01^b^	10.16 ± 0.12^b^	1.25 ± 0.00^b^
LMW	0.11 ± 0.01^a^	0.56 ± 0.00^c^	8.28 ± 0.17^c^	2.00 ± 0.00^c^
Quercetin	4.10 ± 0.01^b^	2.34 ± 0.02^d^	11.73 ± 0.35^d^	3380 ± 150^d^
Rutin	4.08 ± 0.02^b^	1.51 ± 0.05^e^	10.99 ± 0.24^e^	570 ± 40^e^

*Free radicals scavenging activity is expressed as Trolox equivalent antioxidant capacity (TEAC). All antioxidant activities are expressed as mean ± SD (*n* = 3). Means in the same column with different letters (a–e) are significantly different (*p* < 0.05). HMW, high-molecular-weight fraction; MMW, medium-molecular-weight fraction; LMW, low-molecular-weight fraction.*

Scavenging of reactive oxygen and nitrogen radicals is one important mechanism of action by antioxidants. The efficacy of antioxidants in inhibiting lipid peroxidation and thwarting oxidative damage is highly dependent on their free radical scavenging ability (Soobrattee et al., 2005). The DPPH^•^, ABTS^•+^, and ${\text{O}}_{2}^{•-}$ scavenging activities of the fractions were determined by comparing the percentage inhibition of the free radicals to Trolox in terms of TEAC. With the exception of ${\text{O}}_{2}^{•-}$ scavenging assay, there is a strong positive correlation in a dose-dependent manner between the free radical scavenging activities of the fractions and their phenolic and terpenoid contents. This is not surprising, as phenolics and terpenoids such as carotenoids are secondary metabolites that play an important role in the defense against free radicals and thus act as antioxidants, as previously reported (Grassmann, 2005; Surveswaran et al., 2007). The total phenolic and terpenoid contents of the fractions are in the following order: LMW > MMW > HMW (**Table 1**), thus rendering LMW the strongest radical scavenger and reducing agent followed by MMW and HMW (**Table 3**). However, these fractions were found to possess weaker antioxidant activities than the crude cold water extract of the sclerotia. The ability of LMW to reduce ferric ions and scavenge DPPH^∙^ was 4- and 8-fold weaker, respectively, when compared to the sclerotial cold water extract as previously reported (Yap et al., 2013). As for the HMW and MMW, DPPH^∙^ scavenging activity was not detected at all. In fact, the sum of phenolic compounds of these three fractions (18.27 mg GAE/g) was found to be significantly lower compared to the crude cold water extract of 28.23 ± 0.50 mg GAE/g extract (Yap et al., 2013). Thus, consumption of whole *L. rhinocerus* sclerotial powder may be more beneficial as an antioxidant supplement due to the synergistic effect of bioactive compounds in the sclerotia (Machana et al., 2012; Martins et al., 2013).

**FIGURE 4:**
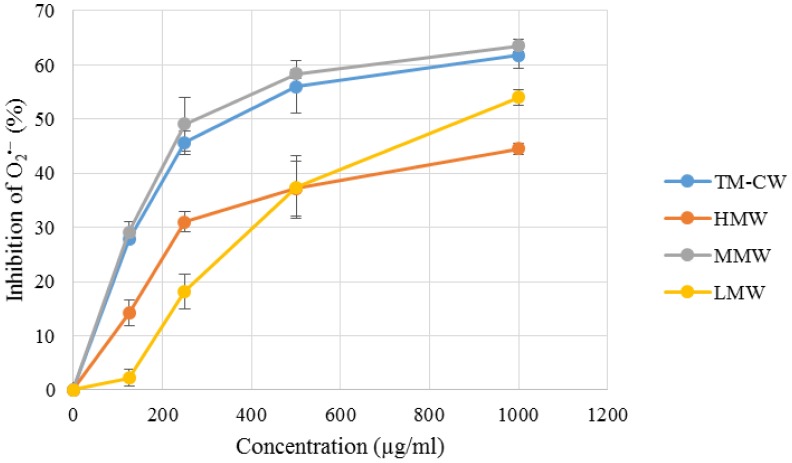
Superoxide anion radical (${\text{O}}_{2}^{•-}$) scavenging capacity of *L. rhinocerus* TM02 sclerotial cold water extract and its Sephadex^®^ G-50 fractions. The scavenging activity was determined by measuring their inhibitory effects on the absorbance of ${\text{O}}_{2}^{•-}$ at 570 nm. Values are expressed as percentage inhibition (mean ± SD, *n* = 3). TM-CW, *L. rhinocerus* TM02 sclerotial cold water extract; HMW, high-molecular-weight fraction; MMW, medium-molecular-weight fraction; LMW, low-molecular-weight fraction.

It is interesting to note that MMW exhibited the strongest ${\text{O}}_{2}^{•-}$ scavenging activity, with potency comparable to that of rutin (**Figure 4** and **Table 3**). Superoxides are continuously produced in all living organisms during metabolism and are biologically toxic to cellular components. Excessive generation of ${\text{O}}_{2}^{•-}$ plays a role in carcinogenesis and induces a pro-inflammatory state in many diseases (Lopez-Lazaro, 2007; Li and Zhou, 2011). When compared with Trolox, ${\text{O}}_{2}^{•-}$ scavenging activity of MMW was 10.16 ± 0.12 mmol TE/g and thus with higher potency than the cold water extract itself (9.90 ± 0.09 mmol TE/g) (Yap et al., 2013). This may indicate that complex polysaccharides and secondary metabolites such as phenolics and terpenoids which are abundantly present in HMW and LMW may not play a significant role in the potent ${\text{O}}_{2}^{•-}$ scavenging activity of *L. rhinocerus* sclerotial extract. The ability of MMW to effectively scavenge ${\text{O}}_{2}^{•-}$ suggests the presence of compound(s) with superoxide dismutase-like activity in the fraction (Nishikimi et al., 1972) or perhaps, the various subtypes of SODs identified in *L. rhinocerus* genome–transcriptome earlier could have been extracted to MMW based on their molecular weight range. In fact, at least two isoforms of manganese SODs (Mn-SOD) at 20 kDa were previously identified in the sclerotium of *L. rhinocerus* by two-dimensional gel electrophoresis coupled with MALDI- and LC- MS analyses (Yap et al., 2015b), further strengthening our hypothesis.

### Glycation Inhibitory Activities of the Sephadex^®^ Fractions

Antioxidants have been shown to protect against glycation-derived free radicals (Elosta et al., 2012; Ramkissoon et al., 2013). In view of the established positive correlation between glycation inhibitory activity and antioxidative potency; the anti-glycation activities of the Sephadex^®^ G-50 fractions were further investigated, as all fractions exhibit antioxidant potency to a certain degree. To date, there is no universally accepted method for AGE measurement owing to their multiplicity and structural heterogeneity (e.g., fluorescent vs. non-fluorescent). The fluorescent AGEs include vesperlysines, Pent, lysyl-pyrropyridine, crossline, fluorolink, and argpyrimidine (Sero et al., 2013) while CML is a well-known example of non-fluorescent AGEs (Pia De La Maza et al., 2012). Since different methods are used for AGE measurement, data comparison between laboratories is often not possible. The simple and fast fluorimetric assay is commonly used to measure fluorescent AGEs regardless of their limitations. The assay may give false-positive results when applied to natural extracts containing fluorescent products and aberrant negative values may be obtained when AGE formation is assessed by arithmetic subtraction of the extract’s fluorescence from those of the extract/AGE mixture (Matsuura et al., 2002). On the other hand, methods that are used for specific AGE detection include high-performance LC, ELISA, and immunohistochemistry (Singh et al., 2001). These assays are costly and deemed unsuitable for rapid screening. In this study, fluorometric assay and an OxiSelect^TM^ AGE ELISA Kit were used to widen the spectrum of AGE detection.

**Table 4** shows the anti-glycation potential of the fractions. HSA-glucose assay was used to evaluate the fractions’ ability to inhibit the glucose-mediated protein glycation. The ability of the fractions to reduce glucose-induced AGE-derived fluorescence decreased in an order similar to their secondary metabolite content and antioxidant potential: LMW > MMW > HMW, suggesting the fluorescent AGE-inhibitory mechanism against glycation was at least partly due to the direct interaction of phenolics and terpenoids with oxidative free radicals generated during glycoxidation (Wu and Yen, 2005). However, these fractions showed weaker overall glycation inhibitory activities than the crude cold water extract and indicate synergistic effects of compounds.

**Table 4:** Inhibitory effects of the Sephadex^®^ fractions on AGEs formation *in vitro*, induced by glucose and methylglyoxal.

	Glucose-induced AGEs (IC_50_^a^, mg/ml)		Methylglyoxal-induced AGEs (% inhibition^b^)
	Fluorescent AGEs	CML, Pent, others	Fluorescent AGEs
AG	0.12	0.52	94.09 ± 0.10^c^ (IC_50_ = 0.34 mg/ml)
TM-CW	5.30	0.02	27.79 ± 1.00^d^
HMW	>10.00	0.002	13.18 ± 0.54^e^
MMW	7.70	0.001	16.43 ± 1.19^f^
LMW	5.20	4.00	38.03 ± 0.68^g^

*Fluorescent AGEs were quantified at 445 nm (λ_exc_ 370 nm). CML, Pent, and other AGE structures were measured using ELISA kit. ^a^Half maximal inhibitory concentration (IC_50_) was determined from concentration-response curve of two independent experiments. ^b^Percentage AGEs inhibition of sample was calculated at 5 mg/ml (mean ± SD, n = 2). Means in the same column with different letters (c–g) are significantly different (p < 0.05). CML, N𝜀-(carboxymethyl)lysine; Pent, pentosidine; AG, aminoguanidine hydrochloride; TM-CW, L. rhinocerus TM02 sclerotial cold water extract; HMW, high-molecular-weight fraction; MMW, medium-molecular-weight fraction; LMW, low-molecular-weight fraction.*

Methylglyoxal is a vital precursor of AGEs *in vivo* which is formed during Maillard reaction (Thornalley et al., 1999). This reactive carbonyl species reacts with the lysine and arginine residues in proteins to produce high molecular weight, cross-linked, fluorescent products (Lee et al., 1998). Earlier, we identified the presence of transcript GME3225_g that encodes for glyoxalase I, a critical enzyme in the detoxification of the toxic glycolytic by-product methylglyoxal and therefore the BSA-methylglyoxal assay was further carried out to investigate the inhibitory effects of the fractions on middle stage of protein glycation. Overall, the fractions exhibited a lower level of middle-stage glycation inhibitory activities. IC_50_ values for all the fractions could not be determined within the range of tested concentrations, impeding the hypothesized anti-glycation role of GME3225_g. Further work is needed to investigate the role of this particular gene in anti-glycation defense. Nonetheless, the inhibition of methylglyoxal-induced fluorescent AGEs by the respective fractions of cold water extract at 5 mg/ml decreased in corresponding order to their secondary metabolite content: LMW > MMW > HMW and a linear regression analysis further showed a positive correlation (*r* = 0.633, *p* < 0.05) between the middle-stage glycation inhibitory activities of the fractions and their antioxidant potential. These findings correlate with the fact that most secondary metabolite-rich anti-glycation agents possessed good antiradical and antioxidant activities (Cervantes-Laurean et al., 2006; Urios et al., 2007), subsequently inhibiting protein modification that may cause AGEs accumulation (Wu and Yen, 2005). Thus, these fractions may have inhibited the methylglyoxal-derived AGE formation by indirectly blocking the conversion of the dicarbonyl intermediate, methylglyoxal, into AGEs; but whether this is through the direct presence of glyoxalase I remains unresolved.

A further investigation shows that the fractions of cold water extract of *L. rhinocerus* TM02 demonstrated a substantially high inhibition toward the formation of non-fluorescent CML and Pent, as quantified by ELISA. Glycation inhibitory activities of these fractions decrease in the following order: MMW > HMW > LMW. It is interesting to note that MMW and HMW exhibited substantial anti-glycation activities when compared with the positive control AG and the cold water extract, with IC_50_ values of 0.002, 0.001, 0.52, and 0.02 mg/ml, respectively. Discrepancy of the fractions’ inhibitory ability toward fluorescent and non-fluorescent AGE formation is not uncommon. Yagi et al. (2012) have reported such a non-linear inhibition in their study on the edible purple chrysanthemum, Chrysanthemum morifolium powder “Enmeiraku” (EPC). EPC extract was shown to be a better inhibitor toward the non-fluorescent CML and Pent with IC_50_ values less than 0.01 mg/ml, while fluorescent AGE formation was inhibited with IC_50_ value of 0.039 mg/ml. It is also worth noting that the aminoguanidine used in their study inhibited the formation of fluorescent AGEs more efficiently than CML and Pent. This observation is in agreement with our study where the IC_50_ values of AG toward fluorescent AGEs and CML, Pent, as well as other AGEs formation as quantified by ELISA were 0.12 and 0.52 mg/ml, respectively. Brownlee reported that the inhibition of mitochondrial ${\text{O}}_{2}^{•-}$ overproduction during hyperglycemia by Mn-SOD prevents an increase in polyol pathway flux, intracellular AGE formation, protein kinase C activation, and hexosamine pathway activity in endothelial cells (Brownlee, 2001); all are associated with hyperglycemia-induced damage that leads to diabetic complications. Therefore, it is reasonable to speculate that the selective inhibitory effects of MMW against CML, Pent, and other AGE-structure formation may be associated with its potent ${\text{O}}_{2}^{•-}$ scavenging activity contributed to by at least a part, if not the whole of the Mn-SOD(s) that is presents in *L. rhinocerus*.

## Conclusion

Pathway annotation of *L. rhinocerus* genome–transcriptome to PlantCyc enzyme database enabled the identification of several interesting transcripts that are related to the biosynthesis of several types of terpenes and terpenoids including carotenoid and abscisic acid, as well as genes that code for glyoxalase I; the key enzyme in the anti-glycation, in addition to CAT-peroxidases and SODs that are able to reduce the oxidative damage in cells through the superoxide radical degradation pathway; suggesting a potential role of *L. rhinocerus* in diabetes management. Further *in vitro* analyses indicated that the MMW isolated from *L. rhinocerus* sclerotial cold-water extract exhibited potent inhibitory effects against CML, Pent, and other related AGE structure formation. The inhibitory activity may be associated with its potent ${\text{O}}_{2}^{•-}$ scavenging activity that could be contributed by SODs that its presence in the mushroom has been validated in our earlier proteomic studies (Yap et al., 2015b). It has been postulated that SODs in the mushroom (which appears to be a main contributing factor to the anti-glycation activity of *L. rhinocerus*) may be destroyed by the low pH and high proteolytic activity in the digestive tract when the mushroom is consumed orally; and that would reduce their bioavailability (Vouldoukis et al., 2004). Nevertheless, recent studies (Vouldoukis et al., 2004) showed that addition of gliadin (a wheat protein) to the SOD supplement offers protection of the enzyme and could increase its bioavailability; while a non-conventional approach is to use topical cream formulation that enable the enzyme to be absorbed into the bloodstream *trans*-dermally (Mizushima et al., 1991). Therefore, drug development of the SOD-containing MMW into a potent glycation inhibitor for diabetes management would be possible by aided coating/encapsulation in order to protect the enzyme during the digestive process for targeted curative effect(s).

## Author Contributions

H-YY performed the experiments and analyzed the data. N-HT, S-TN, C-ST, and S-YF contributed reagents/materials/analysis tools. H-YY, N-HT, and S-YF wrote the paper. All authors conceived and designed the experiments and agreed to be accountable for all aspects of the work in ensuring that questions related to the accuracy or integrity of any part of the work are appropriately investigated and resolved.

## Conflict of Interest Statement

Co-authors S-TN and C-ST are affiliated with LiGNO Biotech Sdn Bhd, which commercialized the tiger milk mushroom. The other authors declare that the research was conducted in the absence of any commercial or financial relationships that could be construed as a potential conflict of interest.

## Footnotes

1 http://www.plantcyc.org

## Supplementary Material

DATA SHEET 1 Assignment of *L. rhinocerus* genes to PlantCyc enzymes database and their respective RPKM values.

DATA SHEET 2 Coding sequences of selected notable genes in *L. rhinocerus*.
